# Abnormal Activity Recognition from Surveillance Videos Using Convolutional Neural Network

**DOI:** 10.3390/s21248291

**Published:** 2021-12-11

**Authors:** Shabana Habib, Altaf Hussain, Waleed Albattah, Muhammad Islam, Sheroz Khan, Rehan Ullah Khan, Khalil Khan

**Affiliations:** 1 Department of Information Technology, College of Computer, Qassim University, Buraydah 52571, Saudi Arabia; s.habibullah@qu.edu.sa (S.H.); w.albattah@qu.edu.sa (W.A.); re.khan@qu.edu.sa (R.U.K.); 2 Institute of Management Sciences (IMSciences), Peshawar 25000, Pakistan; altafkhan@icp.edu.pk; 3 Department of Electrical Engineering, College of Engineering and Information Technology, Unaizah Colleges, Unaizah 56447, Saudi Arabia; sheroz@oc.edu.sa; 4 Department of Information Technology and Computer Science, Pak-Austria Fachhochschule Institute of Applied Sciences and Technology, Haripur 22620, Pakistan; khalil.khan@fecid.paf-iast.edu.pk

**Keywords:** CCTV, CNN, LSTM, lightweight, Hajj pilgrims monitoring, violent activity recognition, crowd monitoring

## Abstract

Background and motivation: Every year, millions of Muslims worldwide come to Mecca to perform the Hajj. In order to maintain the security of the pilgrims, the Saudi government has installed about 5000 closed circuit television (CCTV) cameras to monitor crowd activity efficiently. Problem: As a result, these cameras generate an enormous amount of visual data through manual or offline monitoring, requiring numerous human resources for efficient tracking. Therefore, there is an urgent need to develop an intelligent and automatic system in order to efficiently monitor crowds and identify abnormal activity. Method: The existing method is incapable of extracting discriminative features from surveillance videos as pre-trained weights of different architectures were used. This paper develops a lightweight approach for accurately identifying violent activity in surveillance environments. As the first step of the proposed framework, a lightweight CNN model is trained on our own pilgrim’s dataset to detect pilgrims from the surveillance cameras. These preprocessed salient frames are passed to a lightweight CNN model for spatial features extraction in the second step. In the third step, a Long Short Term Memory network (LSTM) is developed to extract temporal features. Finally, in the last step, in the case of violent activity or accidents, the proposed system will generate an alarm in real time to inform law enforcement agencies to take appropriate action, thus helping to avoid accidents and stampedes. Results: We have conducted multiple experiments on two publicly available violent activity datasets, such as Surveillance Fight and Hockey Fight datasets; our proposed model achieved accuracies of 81.05 and 98.00, respectively.

## 1. Introduction

Hajj is an annual religious gathering for Muslims. Every year, millions of people of different ages, races, and cultures from all over the world come to the Kingdom of Saudi Arabia, specifically Mecca and Medina, to perform the rituals of Hajj and Umrah [1]. This diversified nature of crowds would not have been a reality without the use of modern technologies such as wireless networking, computer vision, spatial computing, data analytics, mobile applications, immersive technologies, and crowd modelling and simulation [2]. The safety of the participants is of primary importance, and it is managed by observing crowd behavior and accurately identifying human activity [3]. Although there is a considerable investment made by the Saudi government towards wireless visual sensor networks with over 5000 cameras installed, along with scalar sensor technologies such as mobile phones equipped with GPS sensors that are used for pilgrim tracking and monitoring, the monitoring of such an event is very challenging due to large numbers of pilgrims attending each year. As a result, stampedes and other violent activities occur, resulting in the loss of precious human lives, as reported in Table 1.

Accurate identification of violent activity is challenging also due to complex patterns, different perspectives, and variance [4]. Researchers have proposed various techniques to efficiently monitor violent crowds during Hajj and Umrah. Dirgahayu and Hidayat [5] have developed a geofencing emergency warning system to help pilgrims in an emergency. They track the pilgrims with their mobile numbers using a built-in GPS unit. Similarly, Mohandas et al. [6] developed a wireless sensor network to monitor pilgrims utilisingutilizing a network that can withstand delay. Every pilgrim has a mobile sensor kit that includes a GPS unit, microcontroller, antennas, and battery. The kit sends identification numbers, longitude, and time to track the user in real time. However, these solutions are based on scalar sensor technology, which has several defects. For example, this technology fails to be of use in certain worship areas and other places where GPS does not work due to signal issues. Secondly, providing each pilgrim with a mobile sensor kit proves to be a very costly affair. Researchers have recently been inspired by computer vision and pattern recognition from the performance of CNN in self-driving cars [7], the smart home [8,9], transportation [10], and other similar fields where it has been applied. Therefore, computer vision and machine learning researchers have also proposed new approaches for efficiently monitoring and managing crowds.

As an instance of computer vision techniques, Khanet al. [11] have developed a system used to detect pilgrims. After training two object detection models from Faster RCNN and YOLO-v3, their performance was analysed. For the dataset, 1339 photos of pilgrims and 952 photos of non-pilgrims were collected from the internet. The authors claim that Faster-RCNN with inception-v2 has achieved 0.59% accuracy with 0.66 F1-score on YOLO-v3, respectively. Likewise, Khan et al. [12] have developed a crowd monitoring system to efficiently manage crowds during Hajj. The crowd’s images were taken from the internet and categorised into normal-crowded, semi-crowded, light, and overcrowded. They have used the two-layer CNN architecture; in the first layer, 32 filters have been used, while in the second layer, 64 filters with 0.5 dropouts have been used, achieving an accuracy of 98%.

Similarly, Hassner et al. [13] have proposed a Bag of Words model framework with handcrafted features called animated blobs that distinguish between combat and non-combat sequences to identify violent crowd activity. Spatial and temporal features were used for feature extraction and classification purposes. Hassner et al. [13] have proposed a descriptor based on the changes in the optical flow magnitude between two frames. The violent stream descriptor that classifies behaviors is based on the Support Vector Machine (SVM).

Similarly, Gao et al. in [14] have proposed a Violent Oriented Flow (OViF) descriptor, which extracts information on the magnitude and direction of movement. The linear SVM is trained on these extracted features to recognise both violent and nonviolent activities. Khan et al. [15] have used the lightweight, optimised MobileNetV1 architecture to identify movies. In [16], the authors have used the sliding window approach and have improved the Fisher vector method for detecting violence. They have achieved an accuracy of 99.5%, 96.4%, and 93.7% in identifying movie violence, violent crowds, and hockey fight datasets. Serrano et al. [17] have recently used Hough Forests with 2DCNN to detect violent activity. Their proposed approach obtained an accuracy of 94.6% in the Hockey Fight dataset. Yu et al. [18] have used Bag of Visual Words (BoVW), feature pooling, and Dimensional Histograms of Gradient Orientation (HOG3D) for violent scenes in videos. The author combines these features based on a Kernel Extreme Learning Machine (KELM) for generalised capabilities. Recently, Habib et al. [19] have proposed a pilgrim’s counter to efficiently monitor pilgrims during Hajj and Umrah in the case of the COVID-19 satiation.

Accurate and precise identification of violent activity in surveillance environments still faces significant challenges such as cluttered backgrounds, different points of view, changing light conditions, different scales, and high computation of CNN models. However, current conventional methods are used to sample algorithms and ergonomic engineering techniques that fail to address these challenges. Recently, methods based on deep learning have solved these challenges to some extent by using recurrent neural network (RNN), long short term memory network (LSTM), bi-directional LSTM, and gated recurrent unit (GRU). However, they do not focus on selecting discriminative features and lightweight models to reduce computational costs. Violent activity is recognised as movements of various human body parts such as legs and arms, etc., in connecting multiple video frames. Therefore, both spatial and temporal information need to be analysed in order to identify violent activity accurately. This paper develops a lightweight framework for identifying violent activity in surveillance during Hajj and Umrah. The following is a summary of the main contributions of the proposed framework:

1. A deep learning assistive framework is developed for the efficient recognition of violent activity by using the visual sensor. In order to efficiently utilise our proposed framework, a lightweight CNN object detector is trained on the pilgrims’ datasets to select only the pilgrims’ prominent frames for further processing.
2. Violent activity is understood as a sequence of motion patterns in the connective video frames. Therefore, both spatial and temporal features are important. For spatial features extraction, since the lightweight MobileNetV2 is transferred for learning violent activity recognition, a lightweight sequential learning LSMT model is proposed for the temporal features extraction.
3. For performance evaluation, we have conducted experiments on two publicly available datasets: Hockey Fight and Surveillance Fight Dataset. Furthermore, an ablation study is undertaken on spatial and temporal features extraction models in order to efficiently recognize violent activities. Finally, the proposed framework triggers an alarm to notify the law enforcement agency to take appropriate action in the case of any violent activity.

The rest of the paper is organised as follows: Section 2 presents the proposed methodology. The experimental setup and evaluation are discussed in Section 3 and Section 4. Section 5 concludes the article, discussing the potential for future investigation.

## 2. Proposed Method

**Figure 1:**
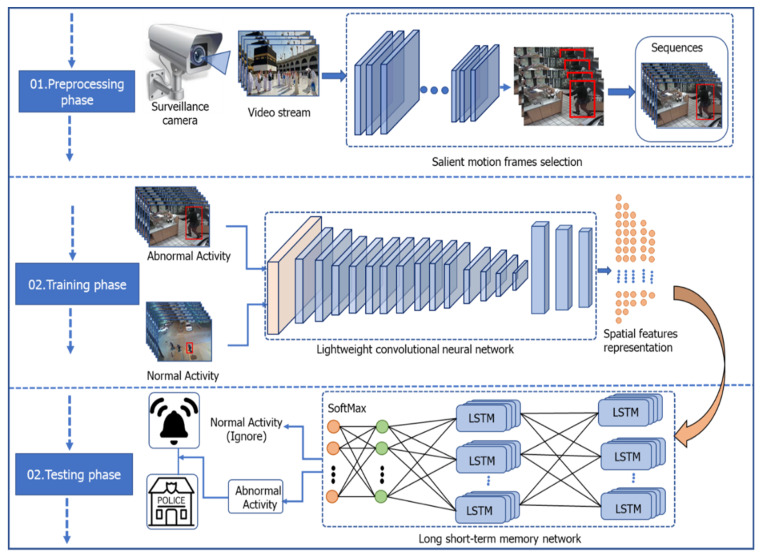
The proposed framework of violent activity recognition.

The proposed framework consists of four steps: In preprocessing, a lightweight CNN pilgrim’s detector is trained on the pilgrim’s dataset to select only specific video frames for further processing. In the second step, a lightweight CNN object detector is developed to extract spatial features. Next, an LSTM model is designed to learn the spatio-temporal features for the accurate recognition of violent activity. In the last step, the proposed framework generates an alarm to inform the law enforcement agency to take appropriate action in the case of violent activity. The complete flow of the proposed framework is shown in Figure 1.

### 2.1. Preprocessing Phase

In this step, the goal is to efficiently utilise the resources of our proposed system by extracting salient pilgrim’s frames from CCTV cameras. Generally, the surveillance system consists of several interconnected cameras to cover a target area for effective surveillance. Processing every video frame is usually not necessary, since very few scenes (such as pilgrims walking) are essential for understanding human activity. There must be some kind of movement of the pilgrims that aids recognition of the abnormal activity. The existing methods used computational techniques that cannot be used on resource-limited devices such as Raspberry Pi or standard CCTV cameras [20].

**Figure 2:**
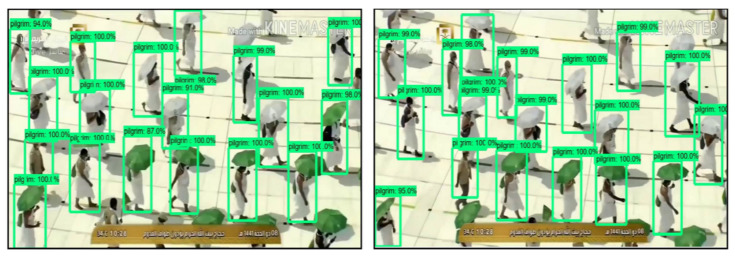
Visual representation of YOLOV4 model on pilgrim’s dataset.

Moreover, the current object detectors are trained on the data of a general class object, effectively detecting pilgrims in surveillance videos. Therefore, we have trained a lightweight CNN object detector such as the tiny YOLO-v4 [21] on a pilgrim’s dataset in order to efficiently detect them in surveillance videos and to pass information on for further processing. Figure 2 shows the input video captured by the surveillance camera during Hajj and Umrah. These videos then provide preprocessing steps to detect whether the frames consist of the pilgrim or not. If there are no pilgrims, then the preprocessing step discards the frame. At the same time, if there are pilgrims, then these video frames are fed into a sequential learning model in order to identify violent and nonviolent activity.

### 2.2. Proposed Architecture

Recognising violence during the Hajj and Umrah is challenging due to the variations in intensity, camera views, and complex crowd patterns. Therefore, traditional methods fail to capture the discriminatory features associated with emotions due to differing illumination, scale, posture, perspectives, and complex human body movements during violent activity. Both spatial and temporal features must be analysed to identify violent action efficiently. Hence, we are required to finetune MobileNetV2 to extract robust discriminative spatial features from violent activity. Then, for spatio-temporal features, a lightweight LSTM model is proposed to identify violent activity in challenging environments, which is easily deployable for devices with limited resources. These models are discussed in detail in subsequent sections. 

#### 2.2.1. Spatial Features Extraction

Deep learning is a subfield of machine learning inspired by the human visual cortex [22]. AlexNet has outperformed traditional handcrafted techniques in the ImageNet Large-Scale Visual Recognition Challenge (ILSVRC) [23]. AlexNet performs exceptionally well in the image classification task; researchers from the computer vision community are exploring and using CNN in several problems: segmentation [24], object tracking [25], plant disease recognition [26], chest disease detection [27], activity identification [28], and other similar areas. The main advantage of CNN architecture is the local connection and weight sharing that helps in processing high-dimensional data and extracting meaningful discriminative features. However, it has required high computation; therefore, it is incapable of being used in devices with limited resources. After multiple experiments on AlexNet and VGG-16, we have concluded that these models have required extensive computation. Consequently, we have used MobileNetV2, which is the enhanced version of MobileNetV1 [29], explicitly designed for devices with limited resources. The idea behind MobileNetV1 has been to use depth-wise and point-wise convolution to reduce the computational complexity of the CNN model; on the other hand, in normal convolutional layers, a kernel or filter is applied to all channels of the input image. For example, in the case of a color RGB image, regular convolution is used on all three channels at once, and their weighted sum is calculated. This convolution requires a high computational cost because it extracts features from all channels at once, while in the MobileNetV1 designs, this computational cost is reduced by a novel mechanism. It extracts features from one channel at a time and then finally aggregates the extracted features. This technique overcomes the overall complexity of regular convolutional-based models by paying some performance degradation.

MobileNetV2 still uses deep detachable convolution, but the bottleneck uses residual blocks instead of deep detachable convolutional blocks. In MobileNetV1, the 1 × 1 convolution makes the number of channels smaller. It is also called the projection layer or the bottleneck layer because it reduces the amount of data flow. In MobileNetV2, the first layer is a 1 × 1 expansion layer that expands the data by increasing the number of channels. Another important layer is the bottleneck residual block or residual connection. Their internal processing is similar to that of ResNet architecture. For activation, a ReLU6 is used (min (max (x, 0), 6)) to consider a positive value of up to 6 digits. We kept the convolutional layers with a lower learning rate (10-6) in order to identify violent activity and to extract discriminative features from violent activity data. 

For the extraction of discriminative features, we have discarded the last layers from MobileNetV2 because it is trained on ImageNet 1000 classes dataset. Instead of this, we have added global average pooling layers, after which we added two dense layers with 1024 and 512 numbers neurons. Finally, for the violent and nonviolent class, we have added two neurons with sigmoid activation for the classification. The initial 20 years layers were frozen for the training purpose and only the following 20 layers were trained at a lower learning rate in order to adjust their weights with the violent activity dataset. The updated MobileNetV2 for violent activity recognition is shown in Table 2. 

#### 2.2.2. Temporal Features Extraction

LSTM is the extended form of RNN and is capable of learning temporal patterns in the time series data. LSTM is the solution to the vanishing gradient problem of a simple RNN that cannot learn long-term sequences and loses the effect of initial sequence dependencies. The LSTM includes input, forget, and output gates that help to learn long-range sequence information. Naive RNN takes the input at each step; however, the LSTM module decides whether or not to take the input using a sigmoid layer so that each gate is open or closed. The input and output parameters of the proposed method are given in Table 3.

As shown in Equations (1)–(3), $i_{t}$, $f_{t}$, and $o_{t}\;$ are input, forget, and output gates, respectively.

In (4), g is the recurring unit calculated from the input at the current time, $x_{t}$, and the hidden state of the previous time step $s_{t-1}$. Equation (6) calculates the current hidden state, $s_{t}$, of the LSTM at time t by tanh activation and (5) memory cell $c_{t}$. The memory cell decides the contribution of the previous time step and current input to calculate hidden state, $s_{t}$. The final state of the LSTM network is represented in (8) as $oS_{tN}$ is the final representation of the entire sequence passed through the Softmax classifier for activity prediction.

(1) $$i_{t}=\sigma \left(\left(x_{t}+S_{t-1}\right)W^{i}+b_{i}\right)$$

(2) $$f_{t}=\sigma \left(\left(x_{t}+S_{t-1}\right)W^{f}+b_{f}\right)$$

(3) $$o_{t}=\sigma \left(\left(x_{t}+S_{t-1}\right)W^{o}+b_{o}\right)$$

(4) $$g=tanh\left(\left(x_{t}+S_{t-1}\right)W^{g}+b_{g}\right)$$

(5) $$c_{t}=c_{t-1}\cdot f_{t}+g\cdot i_{t}$$

(6) $$s_{t}=\tanh(c_{t})\cdot o_{t}$$

(7) $$s_{t}^{1}=\tanh(c_{t}^{1})\cdot o_{t}^{1}$$

(8) $$predictions=sofmax\;\left(s_{tN}\right)$$

The authors extracted spatial features from the video using a lightweight CNN network, after which these features were fed as input at time step *t* into the multilayer LSTM. We have forwarded temporal features of a 1-s video to LSTM in 30-time steps for the learning activity sequence. All the details of the proposed model provided in Table 4.

## 3. Experimental Setup

Multiple runs obtained the experimental results for identifying violent activity on the Surveillance Fight and Hockey Fight datasets. The performance of the proposed system network evaluated by different learning rates using different numbers of convolutional layers and different activation functions. The novelty of the proposed system network model has been established by comparing the results with those of the other state-of-the-art models.

The proposed model has been implemented in Python by using TensorFlow with Google’s Keras deep learning framework. The Core i5 CPU evaluates the model’s training process using an NVIDIA GeForce 1070, 8GB GPU, 24GB RAM. The model has been trained over 200 epochs.

### 3.1. Evaluation Matrices

We have used the most common metrics such as precision, recall, and accuracy to evaluate the proposed model. These are represented mathematically in Equations (9) to (12).

(9) $$Precision=\frac{TP}{TP+FP}$$

(10) $$Recall=\frac{TP}{TP+FN}$$

(11) $$Accuracy=\frac{TP+TN}{TP+TN+FP+FN}$$

(12) $$F1-score=2\cdot \frac{Precision\cdot Recall}{Precision+Recall}$$

Here, the term true positive (TP) represents the number of violent activity correctly identified, while false positive (FP) refers to nonviolent or normal activities incorrectly predicted by the model as violent activity. Similarly, true negative (TN) is then used to represent the numbers of the nonviolent activity correctly identified. False negative (FN) is the number of violent activities incorrectly predicted as nonviolent.

### 3.2. Datasets

**Figure 3:**
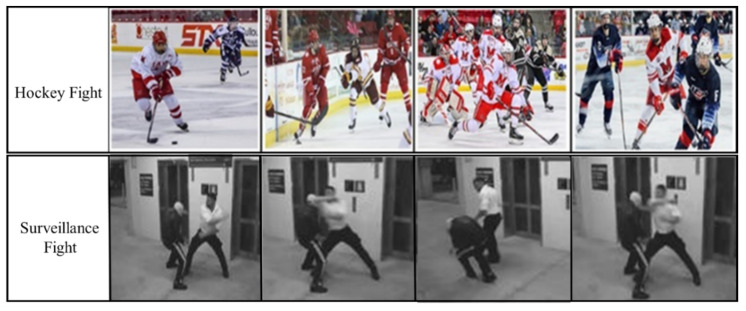
Visual representation of the Hockey Fight and Surveillance Fight datasets.

In order to validate the performance of the proposed model, we have used two publicly available violent activity based Hockey Fights and Surveillance Fight datasets. Their visual representations are shown in Figure 3, while the detailed statistical parameters are shown in Table 5 and Table 6. For training purposes, we have divided these datasets into 80% for training and 20% for validation.

#### 3.2.1. Hockey Fight Dataset

In the first dataset, the authors used Hockey Fight, as presented in Table 5. In this dataset, there are 1000 videos, half of which represent violent fights while the other half represent typical nonviolent activities. All the clips from the fights during a hockey game are collected in the violent class, whereas the normal activities class contains normal scenes. These videos were taken entirely from the National Hockey League (NHL), each video consisting of 50 video frames with a resolution of 360 × 288 × 3.

#### 3.2.2. Surveillance Fight Dataset

This dataset is very challenging considering that all the videos have been taken from YouTube, which respresent real violent scenarios. There are a total of 300 videos of violent and nonviolent category types. Each class includes 150 videos with diverse resolution sizes, where the average resolution is 480 × 360 and 1280 × 720. Their detailed statics are shown in Table 6.

## 4. Results and Discussion

All the experimental results are discussed in detail, concerning experiments with different Learning Rates (LR), different convolutional, and different mechanisms for balancing the trade-off between the model computations. Higher accuracy is maintained at balance.

### 4.1. Spatial Features Results

Spatial and temporal features play a crucial role in the accuracy of violent activity recognition. Thus, experiments are performed on the spatial features of the proposed CNN model. The existing techniques often use retraining weights for different architectures and are ineffective in recognising a complex pattern of violent activity. Other researchers worldwide used the AlexNet and VGG-16 models for various applications, achieving the highest accuracy. Therefore, in the baseline, we have experimented with the AlexNet and VGG-16 models. First, the video frames of the Hockey Fight dataset are resized to dimensions of 277 × 227, and the model is trained over 50 epochs. The Adam optimiser is used with 32 batch sizes. After training, the model achieves 75% accuracy with an F1-score of 0.80 for violent activity, while an F1-score of 0.67 for nonviolent activity was achieved.

Similarly, the VGG-16 model has been trained on the Hockey Fight dataset with 50 epochs. The video frames have been resized to 244 × 244 dimensions. Similarly, to AlexNet, Adam’s optimiser is used with a batch size of 32. After training, VGG-16 has achieved an accuracy of 84% with a score of 0.83 F1. VGG-16 achieves an accuracy 9% higher than AlexNet. The reason for this is that the AlexNet model is a simpler model that cannot extract deep features while VGG-16 is a deep architecture model consisting of 13 convolutions with small filters of a 3 × 3 size. Finally, our proposed model has been trained over 50 epochs using the Adam optimiser. After successful training, the model has achieved an accuracy of 96%, which is 9% higher than that of the VGG-16 and 21% higher than the AlexNet model. Furthermore, a detailed comparative analysis is provided in Table 7.

### 4.2. Sequential Learning Results

From the spatial performance of the proposed model, we have devised further experiments on sequential learning. In sequential learning, the CNN model extracts spatial features, after which they are fed into the LSTM model for spatial-temporal features. From various experiments, we have only reported the best performance model.

#### Ablation Study on Different Sequential Learning Models

In the first experiment, we have designed a sequential model of the seven layers, with a RelU activation function across the network. In the first, second, and third layers, 1024 and 512 numbers of LSTM units are used, respectively. For efficient training, batch normalisation and a 0.1 dropout layer are added between the second and third layers. After this, the features are flattened and fed to 512 fully connected layers in the fourth and fifth layers; then, the fully connected layers are gradually decreased. In six layers, 256 fully connected layers were used and then fed into the sigmoid activation to classify the extracted features into violent and nonviolent activity. The highest accuracy, 0.93, was achieved on a 0.00001 learning rate. Their details are shown in Table 8.

In the second experiment, we used bidirectional LSTM in order to learn about the complex pattern of violent activity efficiently. In the first and second layers, we have used 512 bidirectional LSTM units, the count of which gradually decreased, and in the fourth and fifth layers, we have used 256 and 128 numbers units, respectively. The features are then flattened and passed to 1024 fully connected layers. The number of neurons in the fully connected layers is reduced to 512 in six layers. The extracted features from 512 are directly passed to sigmoid activation, which categorises them into violent and nonviolent activities. Similarly, we have used a different learning rate, and the highest accuracy of 0.935 was achieved by (0.00001) LR, with the details for the same listed in Table 9.

The bidirectional LSTM has not performed very well in comparison to experiment one. Furthermore, it requires several numbers of computation, thus deeming it unsuitable for devices with limited resources and warranting more investigation on the LSMT model. In the third experiment, we have introduced the residual contact between the LSTM layers. In the first and second layers, 512 numbers of LSTM units have been added. Then, the extracted features of the first and second layers are combined in order to enrich the features space. In the fourth layer, 512 neurons are applied to learn from the integrated features effectively. Then, the extracted features are fattened with 512 numbers of fully connected layers. The fully connected layers are reduced into 265 neurons in the last layer and fed into the Softmax classifier for classification. Their details are shown in Table 10, where the highest (0.92) accuracy was achieved on (0.001) LR.

Due to the remaining connection, their search space increased. Therefore, the residual LSTM could not perform very well. From this clue, we conducted experiments on the lightweight LSTM model, as shown in Table 8. We have applied small numbers of LSTM modules to decrease the dimension of the features, fully connected features, and layers, easily classifying violent and nonviolent activity. In the fourth experiment, 512, 156, and 256 neurons have been used in the first, second, and third layers, respectively. After a flat layer is applied, 512, 256 fully connected numbers were applied to classify the extracted features using Softmax activation. Due to fewer LSTM units and fully connected layers, this model achieved the highest accuracy of 0.935 over 0.0001 LR. The complete performance of the lightweight LSTM model with the Surveillance Fight dataset is shown in Table 11.

The performance of the proposed sequential lightweight model is also validated on the Surveillance Fight dataset. Their detailed statistics are provided in Table 12. The model achieved an accuracy of 0.810526 on the 0.0001 learning rate.

Furthermore, the cross folds k = 5 validation is conducted to verify the performance of the proposed model on the k = 5 fold. Table 13 represents the performance of the proposed model on the Surveillance Fight dataset.

Similarly, the same cross fold k = 5 is used for the Hockey Fights dataset, reporting the accuracy and losses of different folds. Their details are shown in Table 14.

### 4.3. Computational Complexity

**Figure 4:**
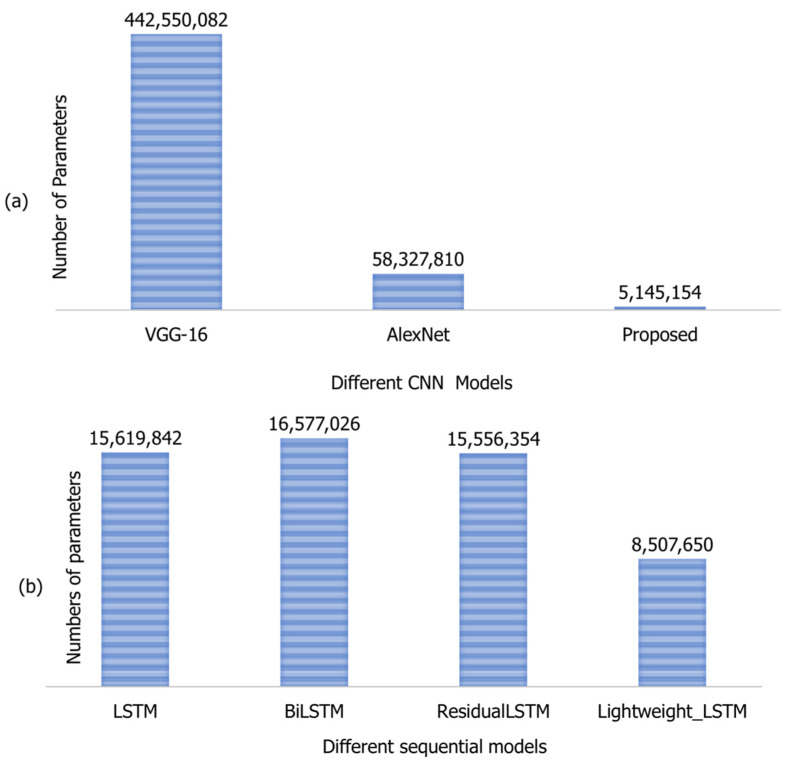
The computational complexity of the models; (**a**) represents the computational complexity of the CNN model, while (**b**) represents the computational complexity of spatio-temporal models.

The computational complexity of the proposed model is evaluated in Figure 4. One of the main advantages of machine learning, particularly CNN, is its exceptional performance on unseen data. However, during testing, it requires a large number of calculations. Therefore, these models have not been successfully developed in devices with limited resources. Researchers are very much interested in transferring CNN application to hardware with limited resources. For computational complexity analysis, several parameters are usually used to analyse the performance of the models. For instance, AlexNet requires 58,327,810 parameters, VGG-16 requires 442,550,082, and the proposed model requires 5,145,154 parameters during testing, as shown in Figure 4a. In sequential learning, the lightweight LSTM model has required 8,507,650 numbers of fewer parameters compared to other models. Their details are shown in Figure 4b.

### 4.4. Comparative Analysis

The performance of the proposed model is evaluated with the existing state-of-the-art methods. Recognising the activity of violence is a challenging task. Various researchers have presented their methods in order to accurately identify violent activity using computer vision. Table 15 summarises the comparative analysis of our proposed approach with the most current methods. For instance, the Bag-of-Words technique has been suggested by [13] to identify violent activity. Their proposed method achieves an accuracy of 82.4% using the Hockey Fight dataset. Likewise, Hassner et al. [14] proposed a statistical descriptor to identify violent activity, achieving an accuracy of 82.4%. The directed violent flow is presented by Gao et al. in [15]. Their method extracts traffic volume and features of AdaBoost from the Hockey Fight violent activity dataset. These features are then categorised by the SVM classifier to achieve an accuracy of 87.50%. Recently, the CNN method has been proposed by Khan et al. [16] for violent activity recognition in movies. The authors claim to have achieved an accuracy of 87.0%.

Moreover, an improved Fisher victor has been proposed by the authors in [17], with 93.7% accuracy. The forest with 2DCNN as proposed in [18] achieved 94.6% accuracy on validation data. For the accurate identification of violent activity, Bag-of-Visual-Words, feature pooling, and Dimensional Histograms of Gradient Orientation (HOG3D) approaches are proposed in [19]. Their method achieved an accuracy of 95.05%. In the following research [20], a two-stream model is proposed. An optical flow feature is extracted in the first stream, while the appearance of invariant features was extracted by Darknet in the second stream. These streams are combined to train the multilayered LSTM model and have achieved 98% accuracy on the Hockey Fight and 74% accuracy on the Surveillance Fight datasets. Apart from the methods mentioned, the CNN method proposed in this paper has achieved a highest accuracy of 96%. Additionally, the sequential model has achieved 98% and 81.05% accuracy on the Hockey Fight and the Surveillance Fight datasets, respectively. Moreover, our proposed approach has required fewer parameters compared to the most recently reported techniques.

## 5. Conclusions

This paper has presented a framework for identifying violent activity in surveillance videos to avoid accidents during Hajj and Umrah. When a violent activity occurs, the system can sound an alarm and notify law enforcement agencies to take the appropriate safety actions required. In order to identify violent activity, we have assessed the performance of our proposed model by using publicly available Hockey Fight and Surveillance Fight datasets. After running multiple experiments, we have achieved 96% accuracy on Hockey Fight and 81.05% on Surveillance Fight datasets, the highest accuracy achieved in comparison with state-of-the-art methods. Furthermore, we have succeeded in balancing the computational complexity of the model suitable for resource-constrained devices. The proposed system can be used in the existing surveillance system to monitor Hajj, especially for crowd monitoring pilgrims on the way to Jamarat.

The existing framework is riddled with a few limitations; we intend to cover them in future research. In this paper, violent activity is recognised from a single view. They cannot cover the full 360° coverage of activity. In the future, we want to recognise violent activity from multiple views to obtain insights on activities. Furthermore, in this research, we have used the publicly available dataset for violent activity. We are currently preparing our own Hajj crowd dataset for violent activity recognition to use in further research. Furthermore, we will explore the performances of different deep learning models, such as two-stream networks, in order to learn both motion and spatio-temporal features efficiently.

## Figures and Tables

**Table 1:** Tragic stampedes during Hajj [3].

Date	Event and Location	Casualties
24 September 2015	Stampede at the junction of streets 204 and 223 in Mina	2110
12 January 2006	Stampede at Jamarat Bridge in Mina	364
1 February 2004	27-min stampede during Jamarat stoning	251
11 February 2003	Stampede at Jamarat in Mina	14
5 March 2001	Stampede at Jamarat in Mina	35
9 April 1998	Stampede/overpass fall off at Jamarat Mina	118
15 April 1997	Fire fueled by high winds in tent city, Mina	343
23 May 1994	Stampede at Jamarat in Mina	270
2 July 1990	Stampede/suffocation in the tunnel leading to Haram	1426
31 July 1987	Security forces break up anti-US demo by Iranian Hajis	402

**Table 2:** The internal details of the proposed MobileNetV2 for activity recognition.

Layer (Type)	Output Shape	Numbers of Parameters	Connected to
global_average_pooling2d	(None, 1280)	0	out_relu[0][0]
Dense	(None, 1024)	1,311,744	global_average_pooling2d[0][0]
Dense	(None, 1024)	1,049,600	dense[0][0]
Dense	(None, 512)	524,800	dense_1[0][0]
Dense	(None, 2)	1026	dense_2[0][0]

**Table 3:** Description of input and output parameters used in the proposed temporal features extraction model.

Variable or Symbol	Meaning
T	Time
$i_{t}$	Input gate
$f_{t}$	Forget
$o_{t}$	Output
g	Recurring unit
$x_{t}$	Input at the current time
$s_{t-1}$	Hidden state of the previous time step
$s_{t}$	Current hidden state
$c_{t}$	Memory cell
$oS_{tN}$	Final representation of the entire sequence
$b_{i}$	Bias

**Table 4:** Detailed summary of the spatial-temporal model for abnormal activity recognition.

Layer (Type)	Output Shape	Numbers of Parameters
InputLayer	(None, 30, 1000)	0
LSTM	(None, 30, 256)	1,287,168
LSTM	(None, 30, 128)	197,120
Batch Normalisation	(None, 30, 128)	512
LSTM	(None, 30, 64)	49,408
Flatten	(None, 1920)	0
Batch Normalisation	(None, 1920)	7680
Dense	(None, 256)	491,776
Dropout	(None, 256)	0
Dense	(None, 128)	32,896
Dropout	(None, 128)	0
Dense	(None, 2)	258

**Table 5:** Description and statistics of the Hockey Fight dataset.

Dataset	Details
Dataset	Hockey Fight [30]
Samples	1000
Resolution	360 × 288 × 3
Violent Scenes	500
Nonviolent Scenes	500

**Table 6:** Description and statistics of the Surveillance Fight dataset.

Dataset	Details
Dataset	Surveillance Fight [31]
Samples	300
Resolution	480 × 360, 1280 × 720
Violent Scenes	150
Nonviolent Scenes	150

**Table 7:** Performance of the proposed sequential learning model on the Hockey Fight dataset.

Model	Category	Precision	Recall	F1-Score	Support	Accuracy (%)
AlexNet	Violent Activity	0.67	0.98	0.80	101	75
	Normal Activity	0.96	0.52	0.67	99	
VGG-16	Violent Activity	0.92	0.76	0.83	101	84
	Normal Activity	0.79	0.93	0.86	99	
Proposed	Violent Activity	0.96	0.97	0.97	101	96
	Normal Activity	0.97	0.96	0.96	99	

**Table 8:** Experimental result of the first sequential model on the Hockey Fight dataset.

LR	Category	Precision	Recall	F1-Score	Accuracy (%)
0.000001	Violent Activity	0.909091	0.9375	0.923077	0.925
	Normal Activity	0.940594	0.913462	0.926829	
0.00001	Violent Activity	0.927835	0.9375	0.932642	0.935
	Normal Activity	0.941748	0.932692	0.937198	
0.0001	Violent Activity	0.909091	0.9375	0.923077	0.925
	Normal Activity	0.940594	0.913462	0.926829	
0.001	Violent Activity	0.843137	0.895833	0.868687	0.87
	Normal Activity	0.897959	0.846154	0.871287	

**Table 9:** Detail summary of the second experiment. A bidirectional LSTM was used to learn the complex pattern of violent activity effectively.

LR	Category	Precision	Recall	F1-Score	Accuracy (%)
0.000001	Violent Activity	0.818182	0.9375	0.873786	0.87
	Normal Activity	0.933333	0.807692	0.865979	
0.00001	Violent Activity	0.936842	0.927083	0.931937	0.935
	Normal Activity	0.933333	0.942308	0.937799	
0.0001	Violent Activity	0.927835	0.9375	0.932642	0.935
	Normal Activity	0.941748	0.932692	0.937198	
0.001	Violent Activity	0.927083	0.927083	0.927083	0.93
	Normal Activity	0.932692	0.932692	0.932692	

**Table 10:** A detailed summary of the tesidual LSTM architecture.

LR	Category	Precision	Recall	F1-Score	Accuracy (%)
0.000001	Violent Activity	0.89899	0.927083	0.912821	0.915
	Normal Activity	0.930693	0.903846	0.917073	
0.00001	Violent Activity	0.908163	0.927083	0.917526	0.92
	Normal Activity	0.931373	0.913462	0.92233	
0.0001	Violent Activity	0.90099	0.947917	0.923858	0.925
	Normal Activity	0.949495	0.903846	0.926108	
0.001	Violent Activity	0.908163	0.927083	0.917526	0.92
	Normal Activity	0.931373	0.913462	0.92233	

**Table 11:** Performance of lightweight LSTM model.

LR	Category	Precision	Recall	F1-Score	Accuracy (%)
0.000001	Violent Activity	0.897959	0.916667	0.907216	0.91
	Normal Activity	0.921569	0.903846	0.912621	
0.00001	Violent Activity	0.919192	0.947917	0.933333	0.935
	Normal Activity	0.950495	0.923077	0.936585	
0.0001	Violent Activity	0.988989899	0.977083333	0.982820513	0.982965
	Normal Activity	0.980693069	0.988461538	0.990731707	
0.001	Violent Activity	0.866667	0.677083	0.760234	0.795
	Normal Activity	0.752	0.903846	0.820961	

**Table 12:** Performance of lightweight LSTM model on Surveillance Fight dataset.

LR	Category	Precision	Recall	F1-Score	Accuracy (%)
0.000001	Violent Activity	0.56338	0.833333	0.672269	0.589474
	Normal Activity	0.666667	0.340426	0.450704	
0.00001	Violent Activity	0.621212	0.854167	0.719298	0.663158
	Normal Activity	0.758621	0.468085	0.578947	
0.0001	Violent Activity	0.8	0.833333	0.816327	0.810526
	Normal Activity	0.822222	0.787234	0.804348	
0.001	Violent Activity	0.527473	1	0.690647	0.547368
	Normal Activity	1	0.085106	0.156863	

**Table 13:** Cross fold k = 5 validation on the Surveillance Fight dataset.

Experiments	Accuracy	Loss
Fold 1	77.89473533630371%	0.6172249455200999
Fold 2	76.31579041481018%	0.9399966472073605
Fold 3	81.05263113975525%	0.5331563221780877
Fold 4	73.68420958518982%	0.8440117999127037
Fold 5	76.59574747085571%	0.6725113670876686
Average scores for all folds:	77.10862278938293	0.721380216381184

**Table 14:** Performance of the proposed model on Hockey Fight dataset on cross folds k = 5 validation.

Experiments	Accuracy	Loss
Fold 1	94.49999928474426%	0.3104947102069855
Fold 2	95.49999833106995%	0.44586579911410806
Fold 3	88.49999904632568%	0.39921369552612307
Fold 4	87.74999976158142%	1.0236860617576167
Fold 5	91.50000214576721%	0.3647297534346581
Average scores for all folds:	91.5499997138977	0.5087980040078983

**Table 15:** Comparative analysis of the proposed model with existing methods.

Methods	Dataset	
	Hockey Fight (%)	Surveillance Fight (%)
Motion Blobs and Random Forest [14]	82.40	--
VIF [15]	82.90	--
ViF, OViF, AdaBoost, and SVM [16]	87.50	--
Fine-Tune MobileNet [16]	87.00	--
Improved Fisher Vectors [17]	93.70	--
Hough Forests and 2D CNN [19]	94.60	--
HOG3D + KELM [20]	95.05	--
Two streams (Optical Flow + Darknet-19) [32]	98	74
Proposed CNN-LSTM model	98.00	81.05
